# Smooth muscle cell fate decisions decipher a high-resolution heterogeneity within atherosclerosis molecular subtypes

**DOI:** 10.1186/s12967-022-03795-9

**Published:** 2022-12-06

**Authors:** Ge Zhang, Zaoqu Liu, Jinhai Deng, Long Liu, Yu Li, Siyuan Weng, Chunguang Guo, Zhaokai Zhou, Li Zhang, Xiaofang Wang, Gangqiong Liu, Jiacheng Guo, Jing Bai, Yunzhe Wang, Youyou Du, Tao-Sheng Li, Junnan Tang, Jinying Zhang

**Affiliations:** 1 Department of Cardiology, The First Affiliated Hospital of Zhengzhou University, No. 1 Eastern Jianshe Road, Zhengzhou, 450052 Henan China; 2 Henan Province Key Laboratory of Cardiac Injury and Repair, Zhengzhou, 450052 Henan China; 3 Henan Province Clinical Research Center for Cardiovascular Diseases, Zhengzhou, 450052 Henan China; 4 Department of Interventional Radiology, The First Affiliated Hospital of Zhengzhou University, Zhengzhou, 450052 Henan China; 5 Richard Dimbleby Laboratory of Cancer Research, School of Cancer & Pharmaceutical Sciences, King’s College London, London, UK; 6 Department of Hepatobiliary and Pancreatic Surgery, The First Affiliated Hospital of Zhengzhou University, Zhengzhou, 450052 Henan China; 7 Medical College, Nanchang University, Nanchang, 330006 Jiangxi China; 8 Department of Endovascular Surgery, The First Affiliated Hospital of Zhengzhou University, Zhengzhou, Henan China; 9 Department of Pediatric Surgery, The First Affiliated Hospital of Zhengzhou University, Zhengzhou, 450052 Henan China; 10 Department of Stem Cell Biology, Atomic Bomb Diseases Institute, Nagasaki University, Nagasaki, 852-8523 Japan

**Keywords:** Atherosclerosis, Smooth muscle cell-based, Single-cell RNA-seq, Cell fate decisions, Molecular subtypes, Heterogeneity

## Abstract

**Background:**

Mounting evidence has revealed the dynamic variations in the cellular status and phenotype of the smooth muscle cell (SMC) are vital for shaping the atherosclerotic plaque microenvironment and ultimately mapping onto heterogeneous clinical outcomes in coronary artery disease. Currently, the underlying clinical significance of SMC evolutions remains unexplored in atherosclerosis.

**Methods:**

The dissociated cells from diseased segments within the right coronary artery of four cardiac transplant recipients and 1070 bulk samples with atherosclerosis from six bulk cohorts were retrieved. Following the SMC fate trajectory reconstruction, the MOVICS algorithm integrating the nearest template prediction was used to develop a stable and robust molecular classification. Subsequently, multi-dimensional potential biological implications, molecular features, and cell landscape heterogeneity among distinct clusters were decoded.

**Results:**

We proposed an SMC cell fate decision signature (SCFDS)-based atherosclerosis stratification system and identified three SCFDS subtypes (C1–C3) with distinguishing features: (i) C1 (DNA-damage repair type), elevated base excision repair (BER), DNA replication, as well as oxidative phosphorylation status. (ii) C2 (immune-activated type), stronger immune activation, hyper-inflammatory state, the complex as well as varied lesion microenvironment, advanced stage, the most severe degree of coronary stenosis severity. (iii) C3 (stromal-rich type), abundant fibrous content, stronger ECM metabolism, immune-suppressed microenvironment.

**Conclusions:**

This study uncovered atherosclerosis complex cellular heterogeneity and a differentiated hierarchy of cell populations underlying SMC. The novel high-resolution stratification system could improve clinical outcomes and facilitate individualized management.

**Supplementary Information:**

The online version contains supplementary material available at 10.1186/s12967-022-03795-9.

## Background

Atherosclerosis (AS) or coronary artery disease (CAD), the most common form of cardiovascular disease, is characterized by the lifelong accumulation and transformation of lipids, smooth muscle cells (SMCs), inflammatory cells, and necrotic cell debris in the intimal space underneath a monolayer of endothelial cells [1, 2]. Despite the declining incidence in several countries, AS remains the leading cause of mortality worldwide [3]. Currently, coronary artery contrast CT and cardio-angiography are broadly but inadequately used to guide clinical management due to the diverse clinical outcomes of patients with the same disease status. CAD is mainly divided into different clinical subgroups according to different clinical manifestations, not adequately encompassing the complex and dynamic behaviour of the disease [4]. The development of molecular classification takes the plunge towards more effective interventions and provides critical insights into AS heterogeneity.

Integration of cell-specific fate mapping and single-cell genomics has proved the advancements in decoding the genomic codes and is widely used to uncover atherosclerotic plaque heterogeneity [5, 6]. SMCs are currently reported to play vital roles in plaque stability and progression via a process of medial proliferation, dedifferentiation, and migration into the intimal lesions in response to stimuli [7]. Human genetic studies have refocused attention on molecules regulating SMC functions as directly causal in CAD [8]. Additionally, the interactions and dynamic variations between SMCs and others are vital for shaping the atherosclerotic plaque microenvironment and further mapping onto heterogeneous clinical outcomes [9]. However, most current therapies for AS target lipoprotein cholesterol, causing little direct impact on SMCs per se [10]. Directly targeting SMC during AS provides therapeutic promise, which could be beneficial or harmful depending on the trajectories of these cells [9].

To tackle this issue, we revealed a roadmap of the cellular fate program in SMCs and a dynamic differentiated state-related signature based on human atherosclerotic plaque single-cell RNA seq integrated with bulk transcriptome data to define potential targets for early intervention better. On this basis, we further introduced an efficient genotyping system, which reflected distinct levels of SMC development and distinguished pathological, clinical, and molecular peculiarities at the individual level. Our findings provided a high-resolution classification system and improved the understanding of AS patient heterogeneity from a developmental perspective.

## Methods

### Available data source and data preprocessing

The human datasets were selected from the Gene Expression Omnibus database under the National Center for Biotechnology Information platform (NCBI), where the method of acquisition and application performed complied with relevant guidelines and policies. The analyzed data we used in this research were mainly acquired from the public database, and hence the need for the local ethics committee approval or patient informed consent was waived. In total, 1074 samples from seven independent public cohorts were enrolled from available databases. A discovery cohort consisted of 195 samples, and another five independent cohorts were used for validation, including GSE20680 (GPL4133; discovery cohort: pbmc samples from 52 atherosclerosis patients with luminal stenosis of ≤ 25%, 56 patients with luminal stenosis > 25% but less than 50%, 87 patients ≥ 70% stenosis in > 1 major vessel or ≥ 50% stenosis in > 2 arteries), GSE20681 (GPL4133; pbmc samples from 99 atherosclerosis patients with ≥ 50% stenosis in ≥ 1 major vessel, 99 patients with luminal stenosis of < 50%), GSE90074 [GPL6480; pbmc samples from 143 stable CAD (sCAD)], GSE59867 [GPL6244; pbmc samples from 46 stable CAD (sCAD) patients, 111 patients on the first day of acute myocardial infarction (AMI), 101 patients after 4–6 days of AMI, 95 patients after 1 month of MI, 83 patients after 6 months of MI], GSE62646 (GPL6244; pbmc samples from 14 sCAD patients, 28 patients on the first day of AMI, 28 patients after 4–6 days of MI, 28 patients after 6 months of MI). The dissociated cells from Diseased segments within the right coronary artery of four cardiac transplant recipients were collected to perform analysis using 10× genomics platform-based single-cell RNA-seq protocol (SRP199578).

The bulk raw data were processed, normalized and corrected using limma and sva packages based on different platforms according to previous studies [11–13]. The Ensembl database was utilized to obtain gene annotations for each probe set. If multiple probe sets correspond to the same gene, the probe set with the highest mean intensity across all samples was retained. The limma package was applied for the differential analysis.

### Single-cell RNA-seq data processing

The quantified single-cell gene expression matrices were analyzed through the Seurat pipeline (Version: 4.1.3). Cells with more than 7.5% of reads from mitochondria genes and less than 500 or more than 3500 genes were removed, while genes expressing in more than 3 single cells were included. Using “FindIntegrationAnchors” and “IntegrateData” functions integrate cells from different samples. The top 2000 variable genes were identified via “vst” selection, considered as the input features for dimensionality reduction using PCA. The first 20 significant PCs determined by jackstraw analysis were incorporated into UMAP analysis for further dimensional reduction and clustering visualization. The findAllMarkers function with “wilcox” method was performed to identify DEGs from the top 2000 variable genes.

### From clustered cells mapping to corresponding cell types

Highly expressed genes of all cell subclusters were used as the potential reference, which was combined with canonical cell-type-specific surface markers derived from CellMarker for comprehensive annotation of cell type. The computational tool scCATCH was also pursued to confirm the inferred cell types in an unbiased fashion. The known cell surface biomarkers of T cell (CD3D, CD3E, CD8A, CD274, CD7), B cell (CD79A, CD79B, MS4A1), monocyte/macrophage (LYZ, CD68, CD14, CD163, FCGR3A), neural cell/Schwann cell (PLP1, S100B), smooth muscle cell (TAGLN, ACTA2, CALD1, MYH11, MFAP4, DCN), mast cell (KIT, HDC), endothelial cell (VWF, CD34, PECAM1, VWF, ICAM2), plasma cell (JCHAIN, MZB1, IGHG3) were selected for annotation of the plaque cell populations.

Based upon previous studies [6, 9, 14–18], the current paradigm was that modulated SMCs can adopt either (1) a pro-inflammatory macrophage-like phenotype characterized by LGALS3, CXCL12, CCL4, KLF4 expression, that could result in plaque destabilization; (2) an extracellular matrix producing “synthetic” SMC synthetic-like phenotype characterized by COL1A1, COL1A2, COL3A1, MGP, FN1, DCN, BGN, LUM, TNFRSF11B, CTHRC1, FMOD, VIM expression, which could contribute to the protective fibrous cap; (3) mesenchymal stem cell (MSC)-like population featured by ENG, NT5E; (4) endothelial cell (EC)-like population featured by VCAN1, CD34; (5) contractile-like population featured by ACTA2, CNN1, MYH11, TAGLN.

### Single-cell RNA-seq data analysis

For cell cycle discrimination analysis and quantification, *Cell Cycle Scoring* function was performed according to previously defined cell cycle-related genes [19]. CellChat pipeline was conducted following the guidelines at https://github.com/sqjin/CellChat. The overall interaction, overall signalling pattern, outgoing/incoming signalling pattern, and ligand-receptor pair were checked in detail step by step.

To analyze the heterogeneity of the differentiated state in SMC lineages, the Monocle2 [20] was carried out to identify the translational relationships among SMC clusters. In summary, genes for trajectory ordering were filtered from the top genes differentially expressed among SMC subclusters using the *differentialGeneTest* function. Following the selection of top 2000 ordering genes through *DDRTress* algorithm (q < 0.001), single cells were projected onto the lower dimensional space reduced from expression profiles and ordered along pseudotime with the *reduceDimension* function. Before identifying the final SMC cell fate decision signature (SCFDS), to minimize the noise-induced error and improve the practicability and operability of biomarkers, features were further screened by integrating multiple algorithms. We retained only molecules as SMC cell fate leader genes that satisfied the conditions as follows:

- genes used for ordering cells along the trajectory from DDRTress algorithm (q < 0.05);
- differential genes among different differentiated states (q < 0.05);
- genes that included in top 2000 variable genes among different SMC phenotypes.

### Subtyping identification through MOVICS

To map differentiation-related gene expression signatures against a series of AS samples, we identified the significant gene modules as the final SMC cell fate leader genes, used for exploring heterogeneity, through the time course analysis by the *Mfuzz* analysis based on bulk level [21, 22]. The CPI and Gaps-statistics were used to estimate the optimum number of clusters, which needs to be small enough to reduce noise but large enough to retain important information. Based on multiple clustering approaches, including ‘SNF’, ‘PINSPlus’, ‘NEMO’, ‘COCA’, ‘LRAcluster’, ‘ConsensusClustering’, ‘IntNMF’, ‘CIMLR’, ‘MoCluster’, we performed each algorithm with default parameters one by one using SMC cell fate decision signature upregulated and downregulated modules. Afterwards, MOVICS algorithm was applied to perform multi-omics integrative clustering and visualization for AS subtyping research [23]. Specifically, under the idea of consensus, we integrated the clustering results derived from different algorithms, ensuring our subtyping robustness.

### Nearest template prediction validation

The nearest template prediction (NTP) is a flexible technique that evaluates class prediction confidence for the single patient [24]. To further test the dependability and stability of clusters, the NTP technique developed in the CMScaller package was leveraged to validate through several cohorts from inconsistent platforms. The subtyping-related highly differential expressed genes were generated as the signature gene list to employ in NTP.

### Exploration of biological interpretability underlying stratification model

Gene sets over-representation analysis (GSORA) based algorithm was implemented to assess whether a particular gene set is over-represented base on the hypergeometric test [25]. Terms were sorted by Z-score. 1000 gene permutations, Z-score cut-off of 1.96, as well as permuted p-value cut-off of 0.05 were adopted. The GSEA algorithm was utilized to decode underlying biometric differences behind ASVS at the bulk- and single-level [26], which was performed through fgsea R package. The number of permutations per gene set was set to 10,000 to yield a normalized enrichment score (NES). Gene sets with a false discovery rate (*FDR*) < 0.05 were deemed statistically significant. The *limma* package was used to decode the similarities and differences between distinct subtypes. In our work, gene lists such as collagen and hallmark pathway signatures were retrieved from MSigDB [27]. The relative activation states of the pathway or specific signature among different subtypes were evaluated by implementing Gene Set Variation Analysis (GSVA) algorithm [28]. It performs a change in coordinate systems, transforming the data from a gene-by-sample matrix into a gene set-by-sample matrix, thereby facilitating the evaluation of pre-defined gene set activities over each sample.

### Cellular heterogeneity estimation

To quantify the relative cell composition fractions in the plaque microenvironment, Cell-type ES was obtained through a gene signature expression-based cell-type enrichment tool xCell (https://xcell.ucsf.edu/) [29]. xCell algorithm analyzes transcripts per million for 64 immune and stroma cell types according to the previously learned information from thousands of pure cell types varying on the sources. This analysis is efficient in reducing associations among closely related cell types, reliably portraying the cellular heterogeneity landscape.

### Statistical analysis

Both two-tailed *p*-values of 0.05 and false discovery rate (FDR) of 0.05 were indicated to be statistically significant in this investigation. Descriptive statistics were calculated using the mean and standard deviation for continuous variables with a normal distribution. Moreover, only continuous variables with an irregular distribution were given a median (range). The correlation between two continuous variables was evaluated through Pearson’s correlation analysis. The Kruskal–Wallis test was utilized to compare the difference among the three groups. The association between categorical variables was examined using the Fisher exact test. R version 4.1.3 software was utilized in all data cleaning, statistical analysis, and visualization.

## Results

### The landscapes of human atherosclerotic plaques revealed by scRNA-seq analysis

**Fig. 1:**
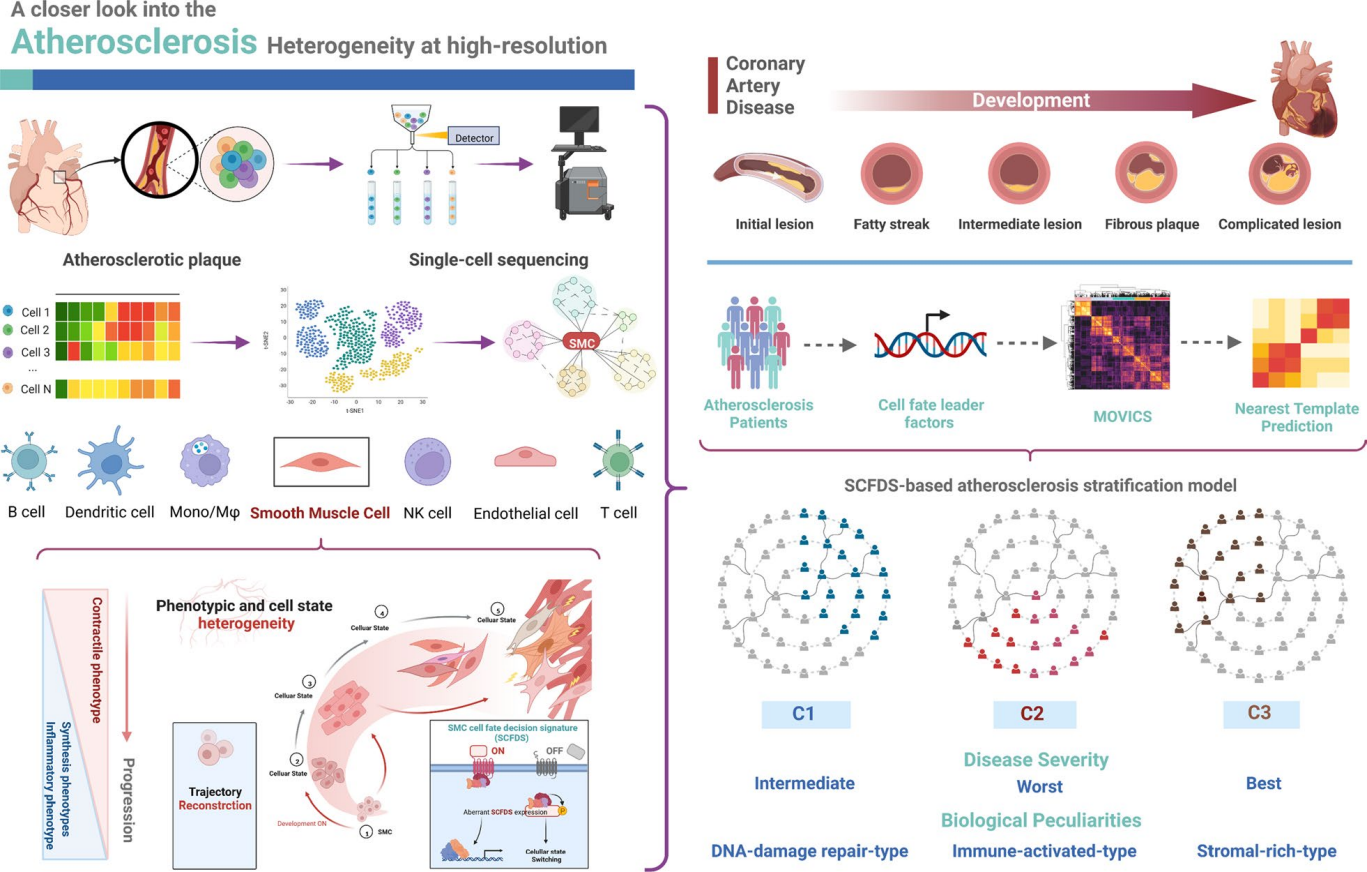
Overall workflow and summary of outcomes in each step

**Fig. 2:**
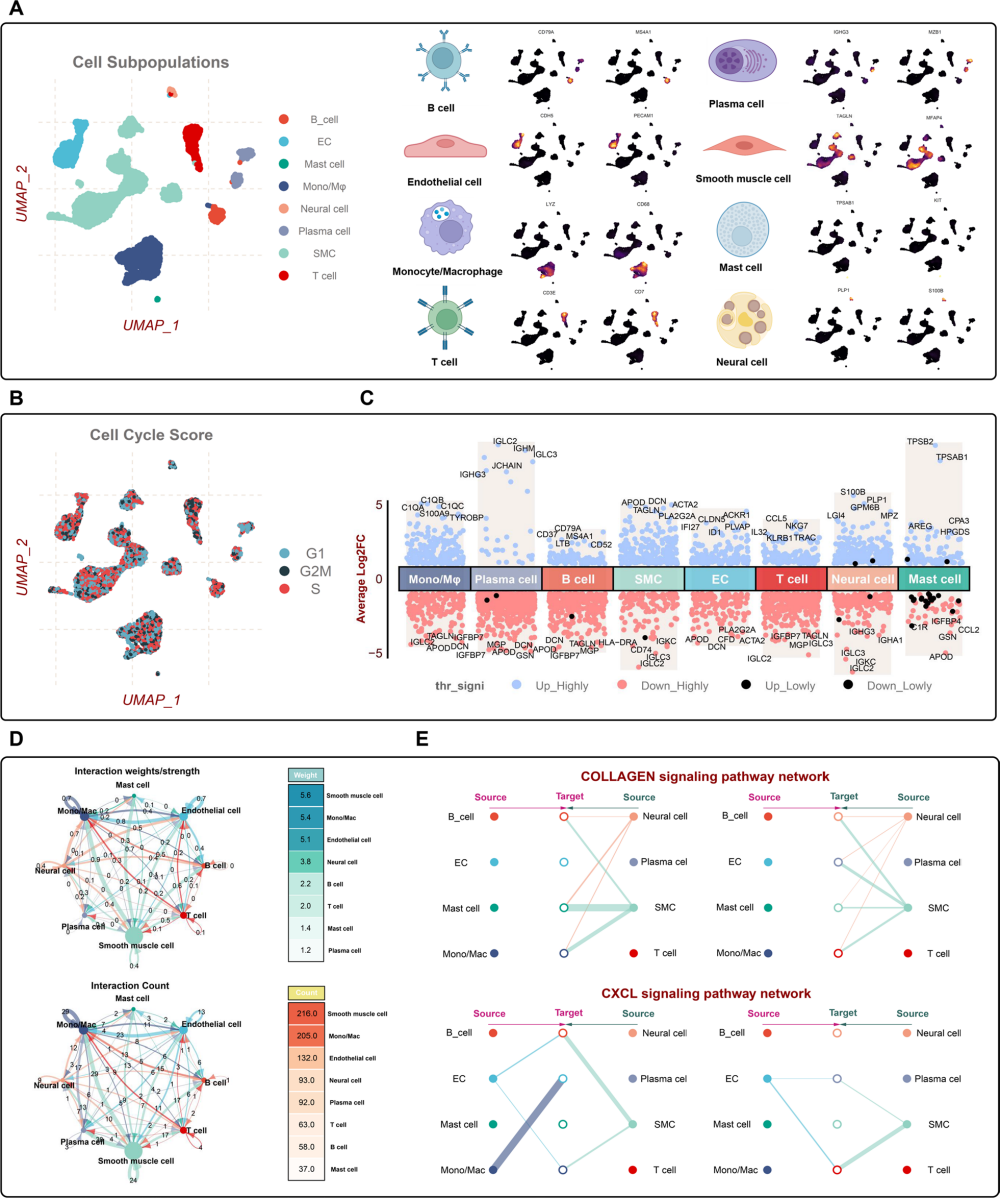
Single-cell RNA-seq profiling revealed cell landscapes derived from atherosclerotic plaques. **A** UMAP plot of 11,756 scRNA-seq cells, with each color-coded for eight cell types (B cells, Endothelial cells, Mast cells, Monocytes/Macrophages, Neural cells, Plasma cells, T cells, Smooth Muscle cells) (left). Expression levels of representative cell type markers on UMAP feature plots (right). **B** UMAP plot of expected outcomes of the cell cycle analysis. **C** Differential expression analysis showing dysregulated genes across each cell type. The adjusted p-value < 0.01 is presented in red, while an adjusted p-value < 0.01 is suggested in black (right). **D** Communication strength-based and number-based interaction networks showing the ligand-receptor interaction prediction focused on SMC and other GBM cells. Solid lines indicated ligand-receptor pairs, with different subpopulations color-coded (left). The heatmap displaying the specific inferred interactions number and strength of all cells in the atherosclerotic plaque (right). **E** The circos plots of the ligand-receptor interaction of Collagen and CXCL signaling pathways among cell populations

A summary of participant flow is provided in Fig. 1. After filtration and quality control for single-cell RNA sequencing data (Additional file 1: Fig. S1), 11,756 cells derived from four patients with atherosclerosis were collected. The UMAP reduction analysis clearly identified 20 clusters and eight cell populations (B cells, Endothelial cells (EC), Mast cells, Monocytes/Macrophages (Mono/Mø), Neural cells, Plasma cells, T cells, SMC) (Fig. 2A and Additional file 2: Fig. S2). The cell cycle score distribution suggested that the majority of the nonimmune cell population, such as SMC and EC, was at S phase, significantly facilitating biological behaviours during AS progression (Fig. 2B). The relative levels of the top differentially expressed genes (DEGs) in each population were presented in the heatmap (Fig. 2C). The ligand-receptor interactions among atherosclerotic plaque cells were determined using the CellChat analysis. SMC cells displayed the highest interactions significantly with other cells based on communication strength and count, which might potentially affect the plaque microenvironment (Fig. 2D). In addition, we found that fibrosis and inflammatory-related pathways, such as collagen and CXCL signalling networks, were strengthened between SMC and immune cells (Fig. 2E). Indeed, during atherosclerosis, SMC most likely contributes to the underlying necrotic core and both the fibrous cap through a development known as ‘phenotypic modulation’, where SMC de-differentiate and proliferates in response to atherogenic stimuli [30, 31].

### SMC lineages’ phenotypic and functional heterogeneity

**Fig. 3:**
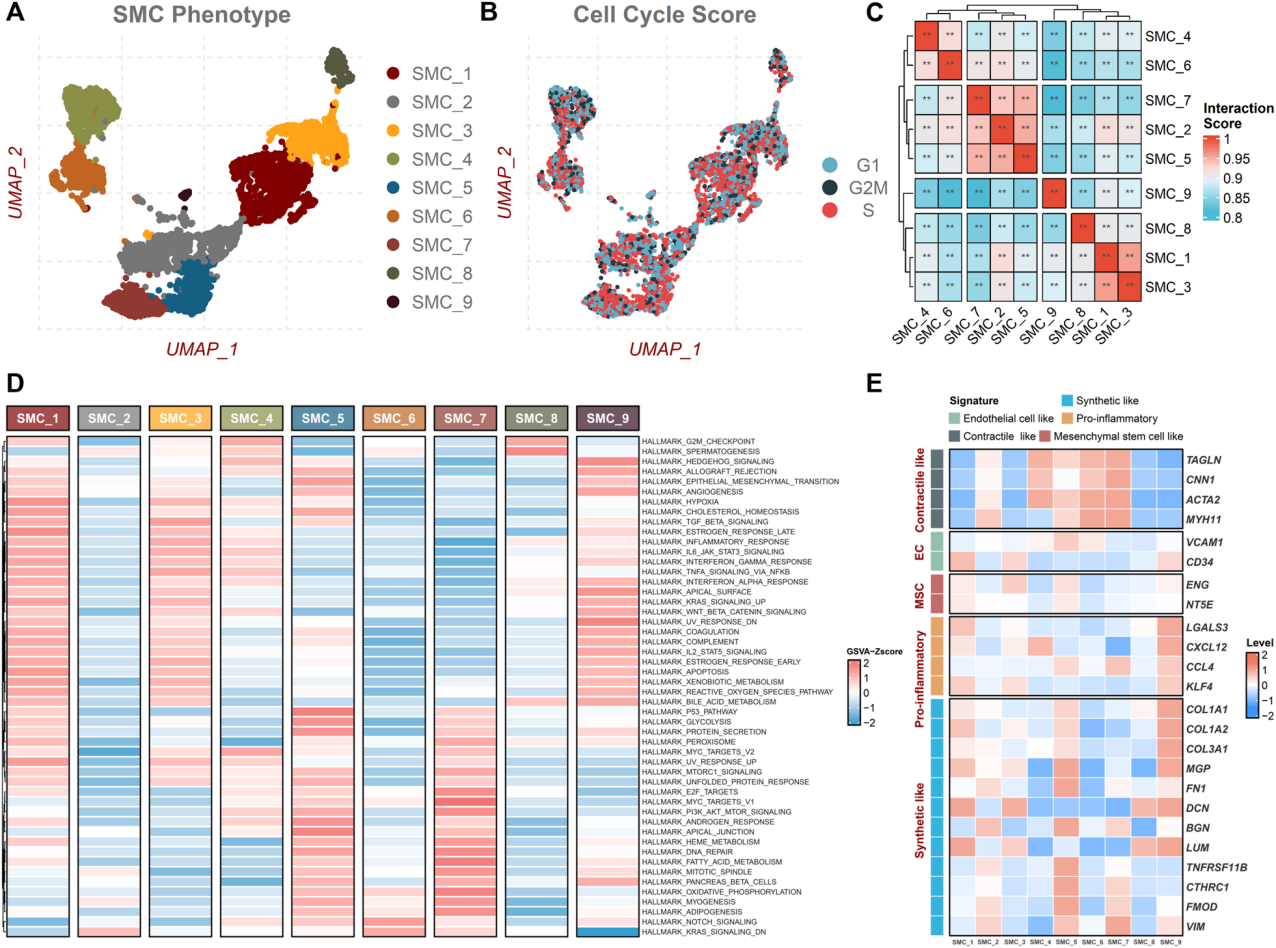
Heterogeneity of SMC in human atherosclerotic plaques. **A** UMAP plot of only SMC, with each color-coded for different subpopulations. **B** UMAP plot projecting the cell cycle states. **C** Heatmap of correlation among different subpopulations. **D** Heatmap representation of the GSVA analysis for each subpopulation based on hallmark gene sets. **E** Heatmap of the average expression of SMC phenotype switching marker genes in each subpopulation

Further analysis was carried out for 5419 SMC extracted from plaque only, where we first performed raw cell clustering through UMAP analysis. All SMCs were segregated into nine major cell clusters (SMC1-C9, Fig. 3A). Next, each SMC single cell was assigned a cell cycle phase score, presenting a higher proliferation score, and the highest proliferating cells were observed in SMC1 and SMC9 subpopulations (Fig. 3B). Moreover, a significant correlation of the most variable genes were identified. SMC2, SMC5, and SMC7 were clustered together, and the close cell–cell interaction between SMC4 and SMC6 was also shown, suggesting a tight relationship involved during AS progression (Fig. 3C). To figure out the molecular mechanism underlying SMC lineages, GSVA analysis revealed that SMC1 involved a wide spectrum of biological contexts, indicating an advanced stage of cell development. SMC3 and SMC4 were found to be implicated in some inflammatory hallmark signalling pathways, such as TNFA signalling via NFKB and interferon-gamma response. Notably, epithelial-mesenchymal transition-related processes were selectively enriched in SMC1, SMC5, and SMC9 (Fig. 3D). SMC4, SMC6, and SMC7, with strong correlations, were characterized by contractile-like classical markers ACTA2, MYH11, CNN1, and TAGLN, related to the mature stage of differentiation. The high levels of mesenchymal stem cell (MSC) markers ENG and NT5E suggested that SMCs undergo a trans-differentiation process during AS. Strikingly, some SMC clusters variably expressed collagen, proteoglycan, and inflammation signatures (Fig. 3E). The comprehensive analyses above indicated polarized populations of distinct phenotypes and functions that emerged in SMC during disease state.

### Trajectory reconstruction revealed SMC cell fate decisions

**Fig. 4:**
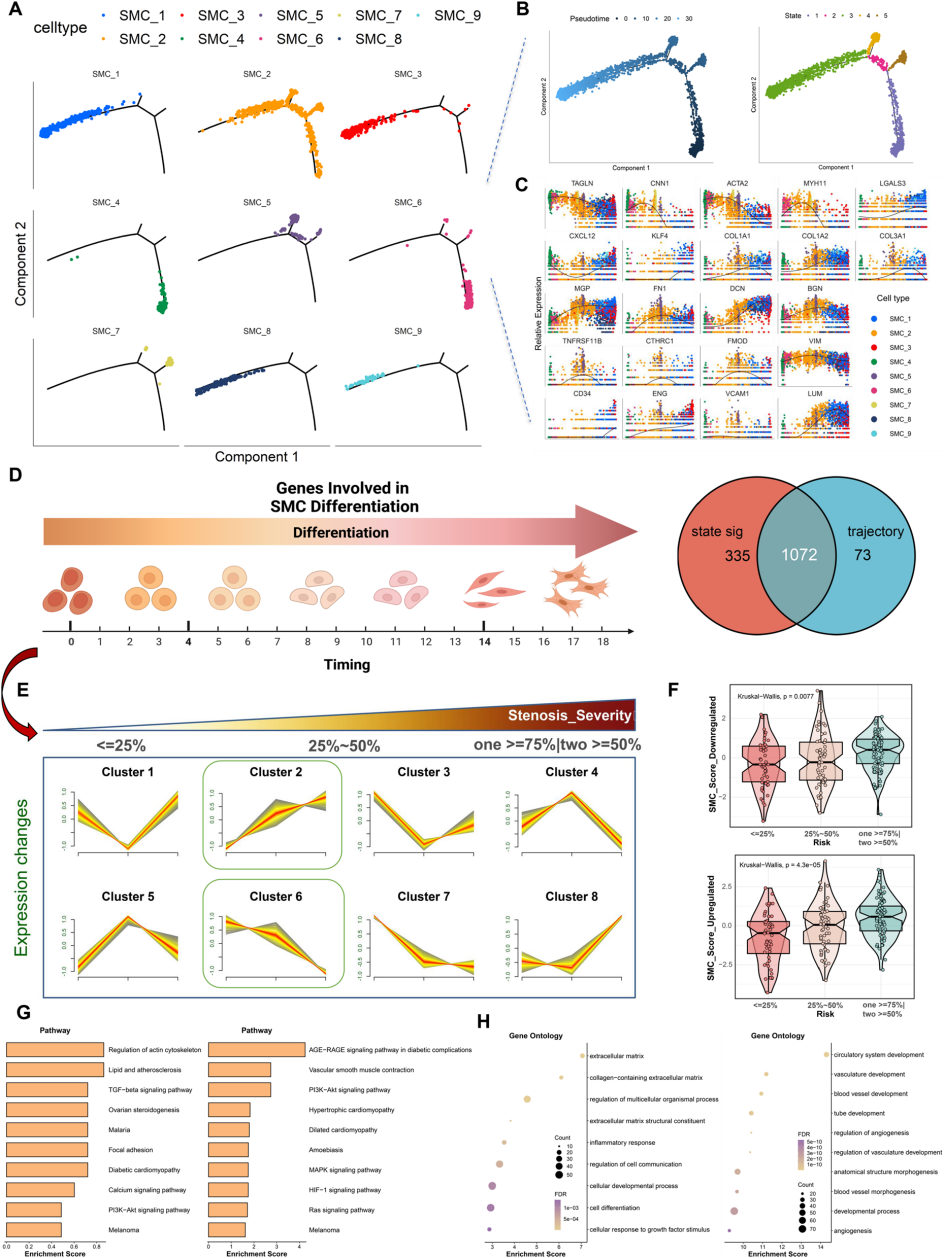
The dynamics of SMC during atherosclerosis progression. **A** Developmental trajectory of SMC with pseudotime, all subpopulations demonstrated in the trajectory. **B** Developmental trajectory of SMC, with color-coded for pseudotime and cell states. **C** Dynamical expression of representative phenotype switching markers plotted as a function of pseudotime, colored by subpopulations. **D** The diagram shows the overlapping of differential genes among cell states and ordering genes, termed cell fate leader genes. **E** Eight gene clusters were obtained via the soft clustering method (Mfuzz) in cell fate leader genes. **F** The violin plot illustrates significant differences in the principal-component 1 score for three risk statuses based on SCFDS. KEGG (**G**) and GO (**H**) enrichment analysis of upregulated (left) and down-regulated (right) SCFDS

To identify the dynamics of SMC cell state over the continuous development process, we performed a pseudotime analysis based on the cell continuous development process by Monocle2 algorithm (Fig. 4A). SMCs bifurcated into vastly five cellular states at two key time points. The SMC4 and SMC6 subpopulations primarily dominated the starting state of the progression trajectory, and mid-state cells were mainly comprised of SMC2, SMC5, and SMC7, which were endowed with high plasticity. SMC1, SMC8, and SMC9 were predominantly in terminal states (Fig. 4B). Evidently, the contractile-like phenotype disappeared gradually with AS progression. The fibroblast-like phenotype marker enriched in the middle-to-end trajectory and upregulated along pseudotime, but sharply decreased sharply at the end of the stage, suggesting the vulnerable features of advanced plaques (Fig. 4C).

Considering SMC phenotype transition reflects AS progression, we identified 1072 genes as SMC cell fate leader genes based on the significant cell state and the trajectory ordering gene set (Fig. 4D and Additional file 3: Table S1). The *Mfuzz* expression profiles of 1072 genes fall into eight groups in their temporal expression dynamics (Additional file 4: Table S2). Modules that were gradually upregulated and downregulated as the AS worsened (Cluster2 and Cluster6) were identified as the SMC cell fate decision signature (SCFDS) (Fig. 4E). SCFDS was subsequently characterized by generating continuous variables SMC_Score_Upregulated and SMC_Score_Downregulated for each sample using principal-component 1 score, which outlined significant differences between AS risks (Fig. 4F). The over-representation analysis (ORA) revealed that the upregulated SCFDS were mainly involved in the extracellular matrix, inflammatory response and TGF-beta pathway, while down-regulated SCFDS in vasculature development and AGE-RAGE pathway (Fig. 4F, H).

### The molecular subtyping of atherosclerosis based on cell fate decision signature

**Fig. 5:**
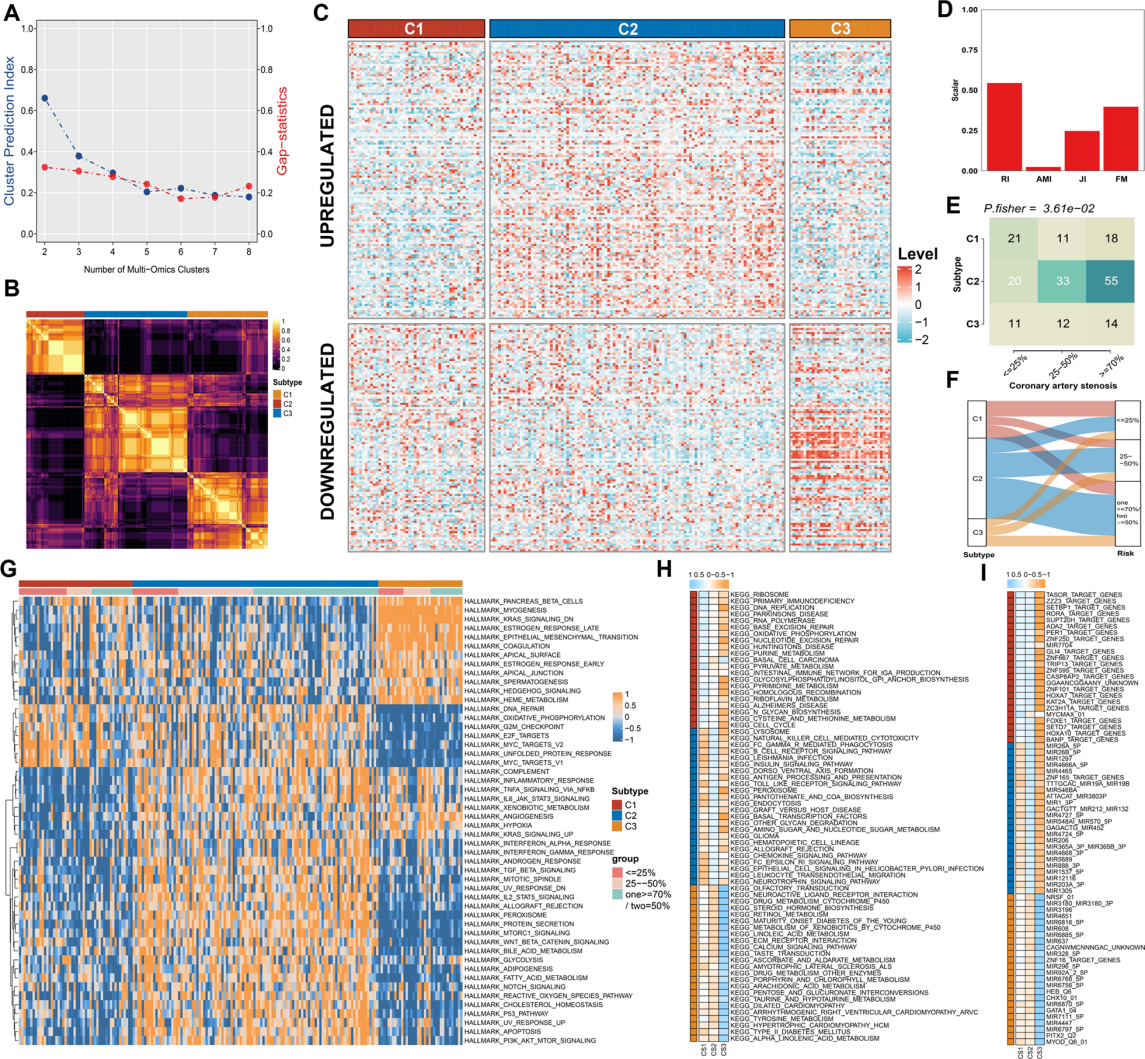
Establishment of three molecular subtypes with heterogeneous illness severity by MOVICS analysis. **A** Determination of optimal cluster number through calculating CPI (blue line) and Gaps-statistics (red line) in AS cohort. **B** Consensus heatmap based on outcomes from 10 multi-omics integrative clustering approaches with subtype number of 3 showing perfect diagonal rectangle. **C** Comprehensive heatmap based on consensus across ten algorithms with upregulated and downregulated SCFDS. **D** Agreement of predicted three subtypes with the severity of coronary stenosis in AS cohort. **E** Comparison of our novel subtype and the severity of coronary stenosis in AS patients. (Fisher exact test; P < 0.05). **F** Alluvial diagram presenting the flow distribution of AS severity between different subtypessubtypes. **G** The distribution of hallmark signatures enrichment score among three subtypes. **H** The distribution of top subtype-specific activated pathways. **I** The most predominant upstream regulators of each subtype

MOVIES is a clustering approach whereby cluster assignments found on multiple data levels are jointly utilized for subtype classification [23]. The optimal subtype number was identified as three following the calculation of the GAP- and CPI-statistics (Fig. 5A). Based on the SCFDS upregulated and downregulated modules, multiple approaches were utilized to decipher the higher-order composition of AS and explore how multiple molecular levels interacted when integrated. MOVICS was then used to seek a stable clustering by hierarchical clustering (Fig. 5B). Notably, SCFDS upregulated along with the AS progression was highly expressed in C2, and downregulated SCFDS showed increased expression in C3 (Fig. 5C). Further, assessing the agreement of novel subtypes with previous stenosis degree of coronary arteries is significant to reflect the robustness of subtyping and determine potential but novel subtypes. Four statistics: Rand Index (RI), Adjusted Mutual Information (AMI), Jaccard Index (JI), and Fowlkes-Mallows (FM), suggested the consistency of the original appraise with the current subtypes as reference (Fig. 5D). To facilitate clinical application, the extent of severity of AS of subtypes was further explored. Patients in C2 exhibited more severe coronary stenosis, while C3 presented a lower risk (P < 0.05) (Fig. 5E).

### Molecular features characterization for SCFDS subtypes

**Fig. 6:**
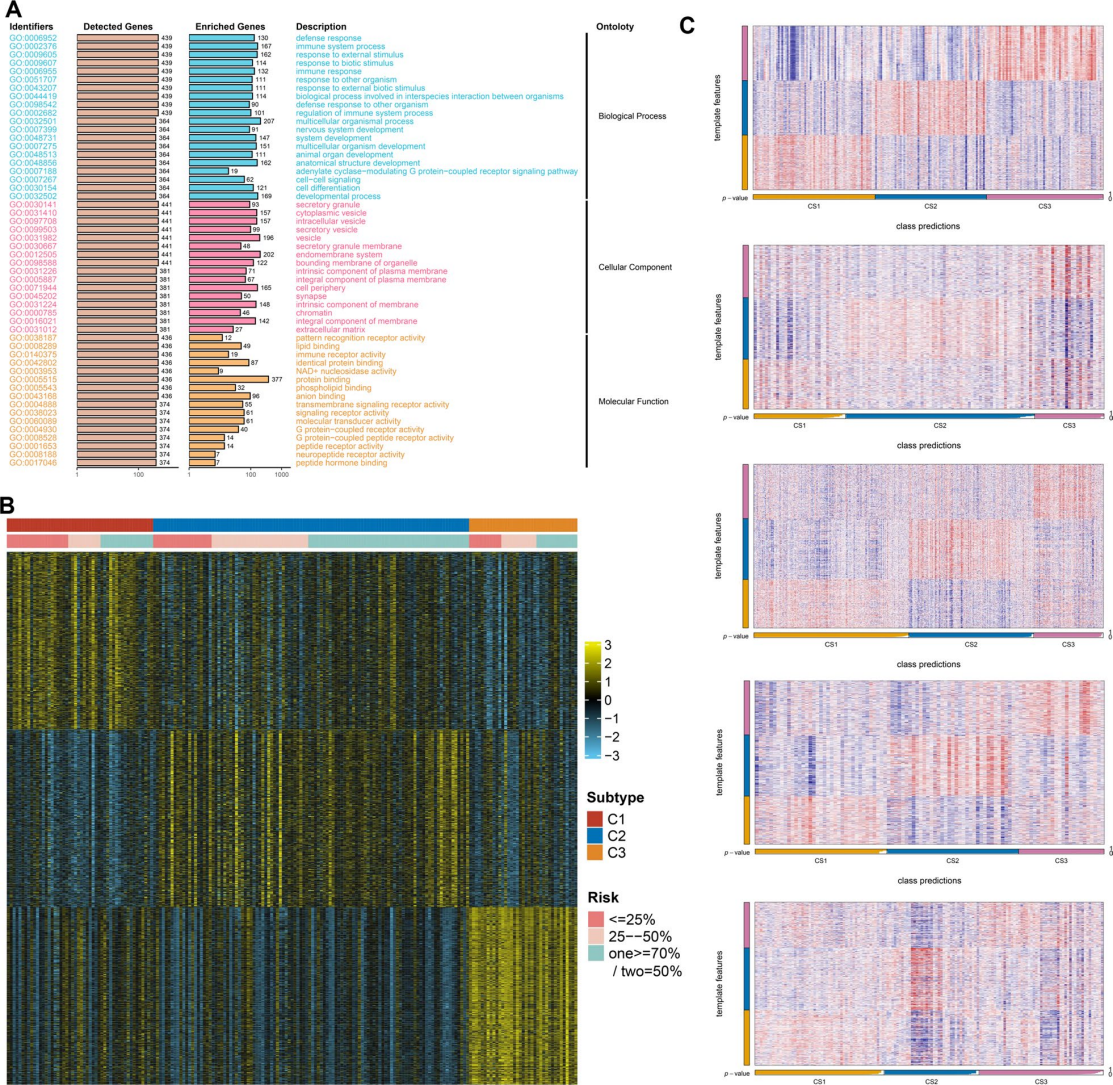
Validation of three heterogeneous molecular subgroups classified by nearest template prediction (NTP). **A** Bar plot of GO pathway ORA results showing significantly different biological functional categories involved in the most high-risk subtype. **B** Heatmap of subtype-specific upregulated biomarkers using limma for three identified subtypes. **C** Heatmap of the template feature expression level between 3 SCFDS subtypes in the GSE20681, GSE21545, GSE59867, GSE62646, and GSE90074 cohorts (panels top to bottom)

To depict the specific biological characteristics of SCFDS-based subtypes, we calculated the enrichment score of each sample based on multiple gene sets. C3, the subtype with the lowest risk of acute coronary syndrome, was explicitly associated with Myogenesis, Angiogenesis and Epithelial–mesenchymal transition hallmark, suggesting high activity in collagen and pro-fibrosis signal pathways. Moreover, the intimal SMC secrete an extracellular matrix consisting largely of collagen to induce a protective fibrous cap against rupture (Fig. 5G) [7]. Similarly, C3 was mainly involved in ECM receptor interaction, Calcium signalling pathway, Dilated cardiomyopathy, Hypertrophic cardiomyopathy HCM and multiple metabolism-related pathways (Fig. 5H). We noticed that C2, where patients exhibited the worst situation, was involved in numerous hallmark signals, suggesting the complexity of the biological mechanism. Hallmarks specifically enriched with C2 were primarily related to inflammation and immune function, especially TNF signalling via NFKB, Inflammatory response, IFN response, TLR receptor pathway, Antigen processing and presentation and Chemokine pathways (Figs. 4I, 5H). Indeed, even in the absence of infection, chronic and low-grade inflammation frequently develops with advanced age, contributing to the progression of AS. Notably, DNA repair, DNA replication, base excision repair, nucleotide excision repair and proliferation-related pathways showed significant selective enrichment in C1, whose severity was considered moderate (Figs. 4I, 5H). DNA damage in SMC has been demonstrated to alter plaque phenotype inhibiting fibrous cap areas in advanced lesions [32]. The failure of DNA repair generated defects in cell proliferation, which in turn resulted in ketosis, hyperlipidemia, and increased fat storage, further promoting AS [33]. Moreover, the most predominant upstream regulators of each subtype were identified in Fig. 5I. The ORA provided a more global understanding of the dysregulated biological characteristics of the most high-risk C2 compared to other subtypes (Fig. 6A). Collectively, we characterized C1 as DNA-damage repair type AS and C2 as immune-activated type AS, whereas C3 was defined as stromal-rich type AS.

### Performance of SCFDS subtypes verified by nearest template prediction

In order to assess the presence of SCFDS-based molecular subtypes in coronary artery disease and myocardial infarction, we applied the Broad Institute’s Nearest Template Prediction (NTP) method. The expression-based classifier for each subtype was generated by identifying the top 300 subtype-specific genes upregulated in each subtype compared with the other subtypes (Fig. 6B). Then, we calculated the distances between each subtype template and the samples to be classified, and the samples were predicted to belong to the subtype with the smallest template distance. Overall, the novel SMC cell fate classification was proven to be reproducible and robust by five independent external cohorts from distinct platforms (GSE26081, GSE21545, GSE59867, GSE62646, GSE90074) (Fig. 6C).

### Assessment of multi-dimensional potential biological implications

**Fig. 7:**
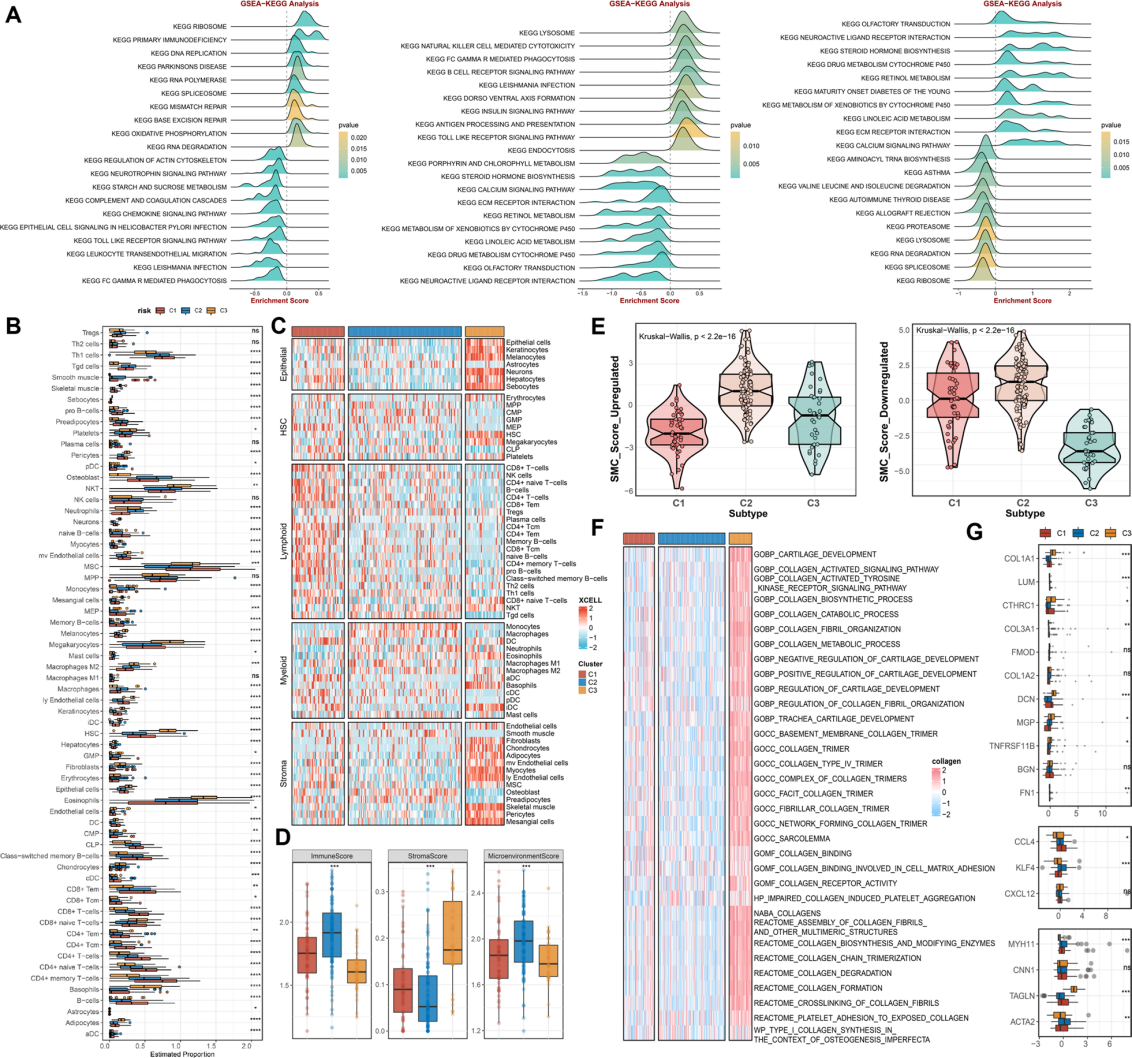
Multi-dimensional analyses uncovered the biological heterogeneity underlying SCFDS subtyping. **A** The ridge plot of the top KEGG terms depicted by GSEA of three subtypes. **B** The boxplot displays the xCell enrichment score of different cell subsets using the xCell algorithm. **C** Distribution of cell abundance in the microenvironment among three subtypes. **D** Distribution difference of the immune score, stroma score, and microenvironment score among three subtypes. **E** The violin plot of significant differences in the principal-component 1 score for three subtypes based on SCFDS. **F** The fibrosis-associated comprehensive pathway and gene set profiles of three clusters. **G** Distribution difference of the SMC differentiation markers expression patterns among three subtypes

Since the phenotypic distinctions could be mirrored by the specific pathways, GSEA analysis was performed to associate each subtype with its corresponding activated signalling. We noticed that except for the pro-inflammatory pathway, infection factors and lysosome, insulin signalling was significantly activated in C2. Moreover, C2 was also characterized by the inhibition of steroid hormone metabolism, calcium signalling and extracellular matrix (ECM) signalling (Fig. 7A). As expected, the individuals with the lowest extent of the severity of coronary stenosis from C3 possessed conspicuous enrichment in steroid hormone and ECM metabolism. C1 patients with a moderate degree of blockage could be regulated by DNA replication, Mismatch repair, and base excision repair (Fig. 7A).

Next, we compared the landscape of cell composition within the AS microenvironment using the xCell algorithm. The outcomes indicated that the most inflammatory cells presented a significantly higher infiltration in C1 and C2.

Monocyte, especially macrophage M1, had an increasing and more significant infiltration profile than other immune cells in C2. Surprisingly, a significantly higher degree of fibroblast and chondrocyte fractions was found in C3 (Fig. 7B, C). Collectively, elevated immune levels and loss of stroma density were present in the C2 microenvironment (Fig. 7D).

The change in SCFDS-based SMC_score_upregulated/downregulated over the development of advanced AS has been demonstrated previously (Fig. 4F). Here, we assessed the SMC_score of each subtype and found C2 was in an advanced stage of AS while C3 was in an early stage (Fig. 7E). Additionally, AS lesions from the C3 subtype were mainly likely to accumulate prominently ECM consisting essentially of collagen, which could give rise to a protective (against rupture) (Fig. 7F). To further explore the SMC phenotypic switch in AS progression, we evaluated the SMC differentiation markers expression patterns in different molecular subtypes. Subtype C2 was characterized by the low level of the fibroblast-like phenotype markers (Fig. 7G), which also decreased sharply at the end of the SMC differentiation stage at single-cell resolution (Fig. 4C). Unsurprisingly, these collagen and proteoglycan genes exhibited high expression in C1. In contrast, the contractile-like SMC phenotype was relatively lost (Fig. 7G).

Collectively, the above mentioned findings revealed a wide biological heterogeneity among AS patient population, which may provide implications for further studies of meticulous management and individual therapies.

## Discussion

Understanding the heterogeneity of AS individuals could facilitate more meticulous management to retard the development of clinically significant CAD and its consequences [4]. Integration of single-cell genomics, cell-specific fate mapping, and human genetics contribute to decoding cell complexity and novel genetic regulation of disease. Here, we leveraged the cellular fate state of SMC to discover the novel heterogeneous subtypes of AS based on the lineage differentiation hierarchy. As previously reported, SMC could partly modulate the stability and progression of atherosclerotic plaque through phenotypic switching in response to atherogenic stressors [15, 31], preferably characterizing the biological patterns of patients. Notably, current data [6, 9] lacks an in-depth analysis of the heterogeneity of SMC populations and the genetic mechanism underlying differentiation in humans. It is necessary to explore key bioactive molecules governing SMC cell fate decisions and develop more precise stratification.

Our results indicated that SMC exhibited the strongest cross-talk with other cells and constituted the core communication position in the human atherosclerotic plaque. One previous animal study has also reported that SMC-derived cells account for a large proportion of cells within AS lesions [34]. We further uncovered the existence of nine distinct SMC phenotypes, characterizing the transcriptional profiles and their functional heterogeneities. Currently, it remains uncertain whether the SMC phenotypic switching presented predominantly atheroprotective or harmful. Along with disease progression, we found that all phenotypes were mapped to five differentiation states and two cellular fates (inflammation-potentiating and ECM-producing). The shift toward a pro-inflammatory fate may serve to destabilize the lesion, while an extracellular matrix-producing fate may contribute to the protective fibrous cap, preventing plaque rupture [15]. In this work, the extent of pro-inflammatory and ECM markers was demonstrated to be significantly upregulated in the mid- and end-stage of the trajectory. Notably, the extent of collagen markers was elevated more intensely in ECM-producing fate compared to the markers in inflammation-potentiating fate. Nevertheless, this finding contrasts the previous report that SMC transdifferentiated predominantly into macrophage-like cells in mouse lesions [35]. Moreover, we also demonstrated that the loss of contractile phenotype contributes to lesion progression. SMC polarization depends on the disease microenvironment and transcriptome landscape during malignant cell fate commitment. In our opinion, SCFDS underlying the programming of the SMC differentiation state was significant to heterogeneous clinical efficacy and also an excellent choice to construct molecular subtypes for AS patients.

To address the lack of an efficient stratification system reflecting distinct levels of molecular differentiation and development of SMC within lesions, the MOVICS clustering algorithm was conducted to establish a novel AS stratification model using SCFDS. The patients were split into three novel molecular subtypes. Considering that the stability and reproducibility of molecular subtypes are fundamental for clinical application, the SCFDS taxonomy was rigorously verified in five external cohorts with distinct platforms. Our subtyping maintained comparable proportions and shared analogical transcriptional in the discovery and validation cohorts.

Furthermore, the SCFDS taxonomy also conveyed clear molecular and biological interpretability, providing a foundation for future risk stratification and personalized treatment decision-making. Briefly, the MOVICS clustering labels were recapitulated as follows.

C1, a DNA-damage repair type, is endowed with elevated base excision repair (BER), DNA replication, nucleotide excision repair, and oxidative phosphorylation status. The coronary stenosis severity of this subtype falls in a range among the others. Thus, further interventions should focus on how to convert C1 into subtypes with better clinical outcomes. We have demonstrated that SMC clusters in the intermediate stage of differentiation also shared such biological characteristics, suggesting a progressed stage in this subtype. Indeed, some human DNA damage syndromes are reported to be associated with premature atherosclerosis [36]. The development of atherosclerotic plaques demonstrated an extensive 8oxoG accumulation, the most abundant DNA damage formed on oxidative exposure [37, 38]. Polymorphisms in some BER enzymes also correlated with MI [39], promoting plaque development or vulnerability. Importantly, Studies have shown that endogenous levels of oxidative DNA lesions in vascular SMCs accelerate plaque development, and correcting the BER defect in SMC alone can markedly reduce plaque formation [40]. For C1 AS, patients are suitable for increased DNA repair and protection against oxidative DNA damage in SMCs, effectively preventing the condition deterioration.

As described above, C2, an immune-activated type, is characterized by stronger immune activation, hyper-inflammatory state, complex and varied lesion microenvironment, advanced stage, and the most severe degree of coronary stenosis severity; more considerations are needed to facilitate outcomes and therapeutic efficacy for patients. Many immune signalling pathways were activated in this subtype, with a large amount of inflammatory cell infiltration, mainly monocytes and macrophages. The macrophages contribute to plaque destabilization by amplifying inflammation, producing proteases, and attacking the fibrous cap [4]. The lysosomal biogenesis process was also enriched in C2, suggesting its non-negligible potential to benefit from macrophage autophagy-lysosome system-based therapy [41, 42]. We also documented that significantly elevated neutrophil counts were predominantly assigned to C2. Neutrophils, although rare in common atherosclerotic lesions, were demonstrated to trigger endothelial erosion through the secretion of matrix metalloproteinases, further accelerating artery thrombosis in this subtype [1, 43]. Our study further also proved that C2 featured by abundant IFN response, indicating C2 patients could be intervened by specific inhibitors antagonizing IFN-related signaling cascades. Indeed, the effect of IFN-gamma on other cytokines further inhibits the synthesis and excretion of ECM and collagen deposition, leading to plaque rupture [44].

C3, a stromal-rich type, is distinguished by abundant fibrous content, a high level of ECM metabolism, and an immune-suppressed microenvironment. Our study indicated that SMC in this subtype was undergoing the transdifferentiation into synthetic’ fibrotic phenotype, increasing the protective fibrous cap thickness [7]. Except for the mild coronary stenotic lesions, C3 commonly exhibits decent clinical outcomes due to the atherosclerotic lesions with thick fibrous caps tend to be more stable than fatty, inflammatory plaque. Thus, further interventions should focus on how to block SMC transition to the pro-inflammatory and dysfunctional phenotype coincident with attenuation of atherosclerotic severity, such as all-trans retinoic acid (ATRA) for the RA signalling activation [9, 45]. Moreover, we also found that C3 was significantly associated with the drug metabolism-cytochrome P450 (CYP) pathway, and CYP1B1 could serve as additional supplements for routine agents [46].

Some limitations of this work should be acknowledged. Due to the bias base on pure computational biology, our study cannot fully recapitulate the diversity of developmental states within SMCs. Further experiments and prospective multicenter studies are still imperative to validate the biological interpretability of SCFDS and support the clinical relevance of novel subtyping from multiple dimensions. All the samples enrolled in this research were retrospective, and a prospective study should be applied to validate the results.

## Conclusions

Our study developed and validated an efficient subtyping system from the perspectives of molecular differentiation and the development of plaque SMCs. The multifariously biological and clinical peculiarities of this novel high-resolution taxonomy contribute to understanding disease heterogeneity and facilitate risk stratification and individuation management for atherosclerosis patients.

## Supplementary Information

**Additional file 1: Figure S1.** Single-cell RNA-seq quality control and preliminary analyses.

**Additional file 2: Figure S2.** From clustered cells mapping to atherosclerotic plaque cell types.

**Additional file 3: Table S1.** Information of SMC cell fate leaders.

**Additional file 4: Table S2.** Information of mfuzz gene modules.

## Data Availability

The datasets presented in this study can be found in online repositories. The names of the repository/repositories and accession number(s) can be found in the article.
