# Supplementary figures and images for: Smooth muscle cell fate decisions decipher a high-resolution heterogeneity within atherosclerosis molecular subtypes
Source: J Transl Med. 2022 Dec 6;20:568. doi: 10.1186/s12967-022-03795-9 (PMC9724432; doi:10.1186/s12967-022-03795-9)

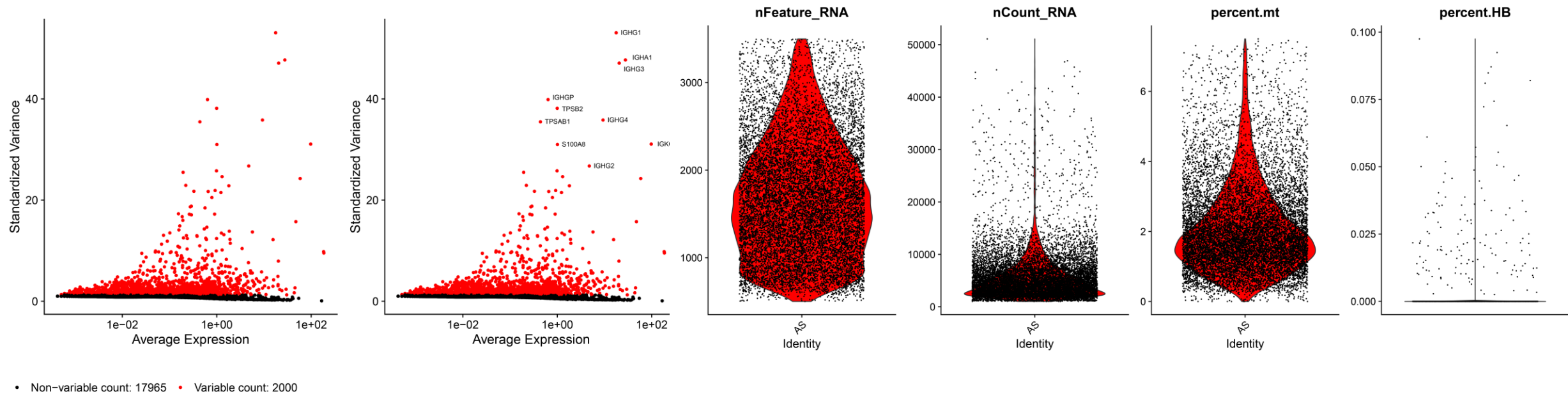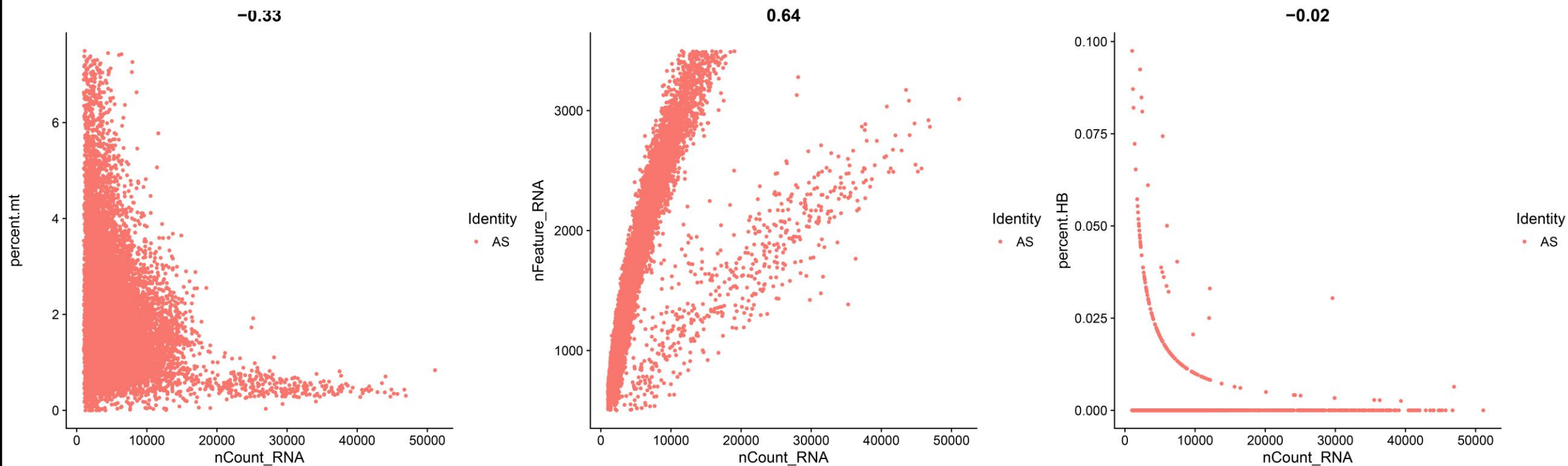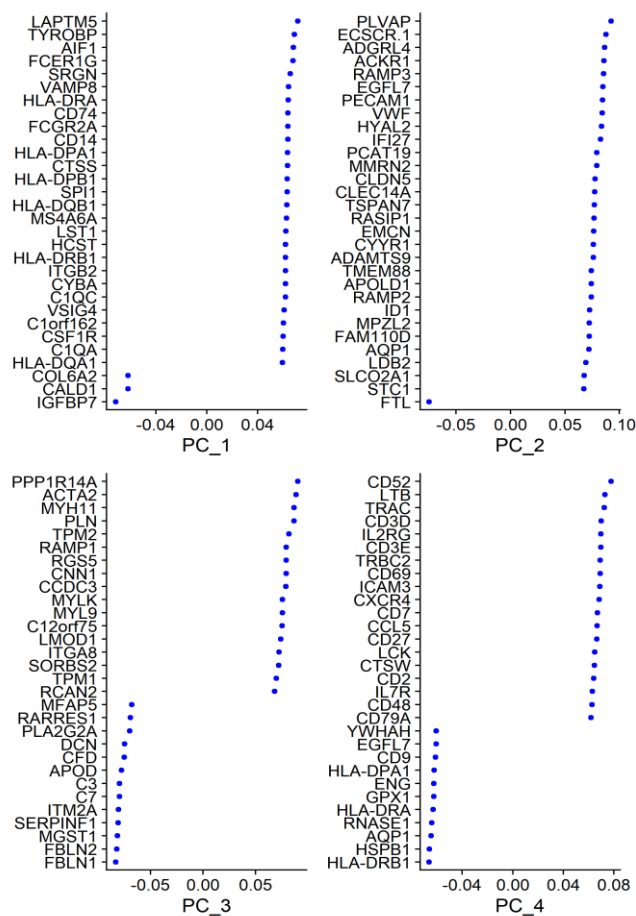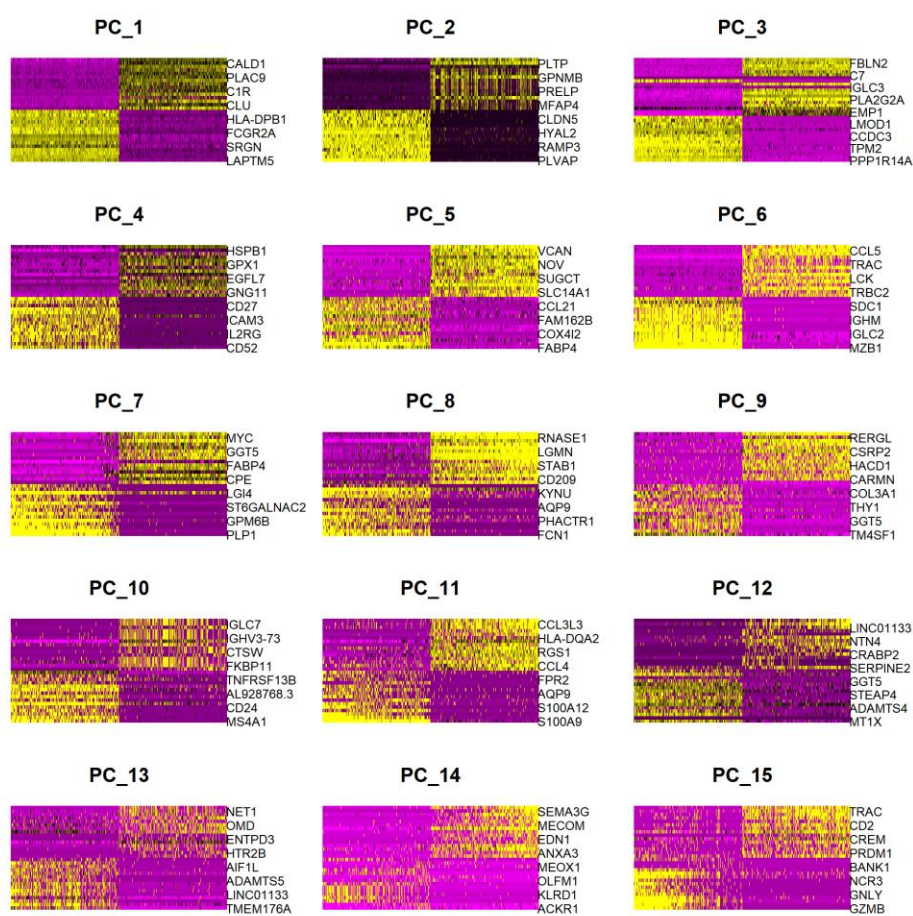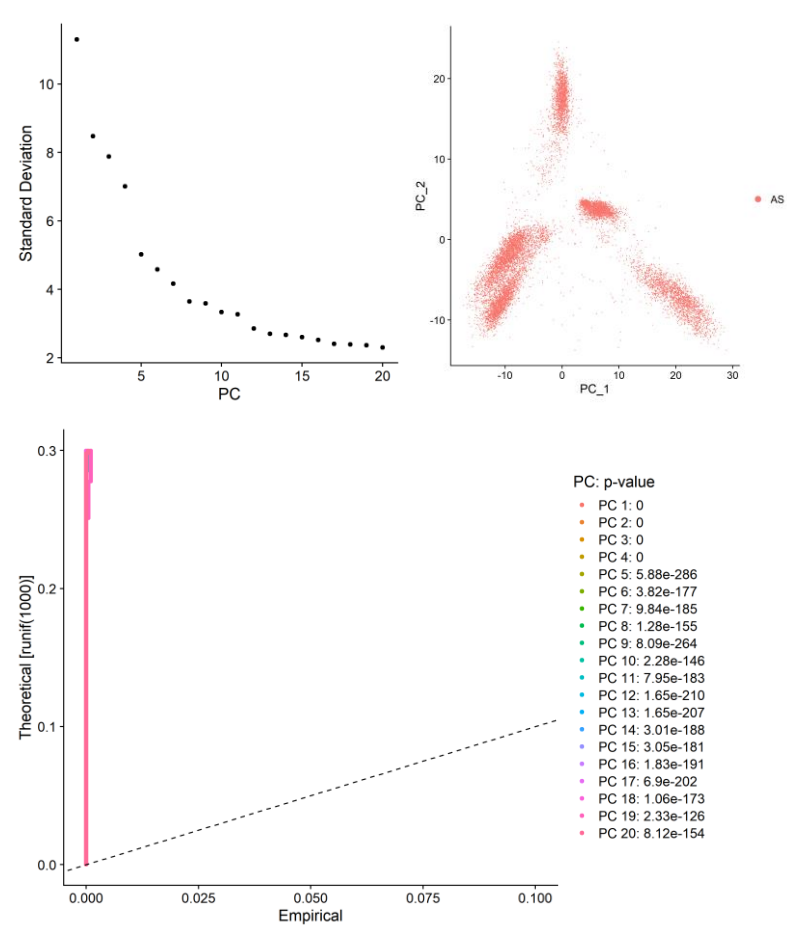

Supplement: Supplementary file 1 — Additional file 1: Figure S1. Single-cell RNA-seq quality control and preliminary analyses. [file 12967_2022_3795_MOESM1_ESM.pdf]
