# Supplementary material for: Smooth muscle cell fate decisions decipher a high-resolution heterogeneity within atherosclerosis molecular subtypes
Source: J Transl Med. 2022 Dec 6;20:568. doi: 10.1186/s12967-022-03795-9 (PMC9724432; doi:10.1186/s12967-022-03795-9)
Supplement: Supplementary file 2 — Additional file 2: Figure S2. From clustered cells mapping to atherosclerotic plaque cell types. [file 12967_2022_3795_MOESM2_ESM.pdf]

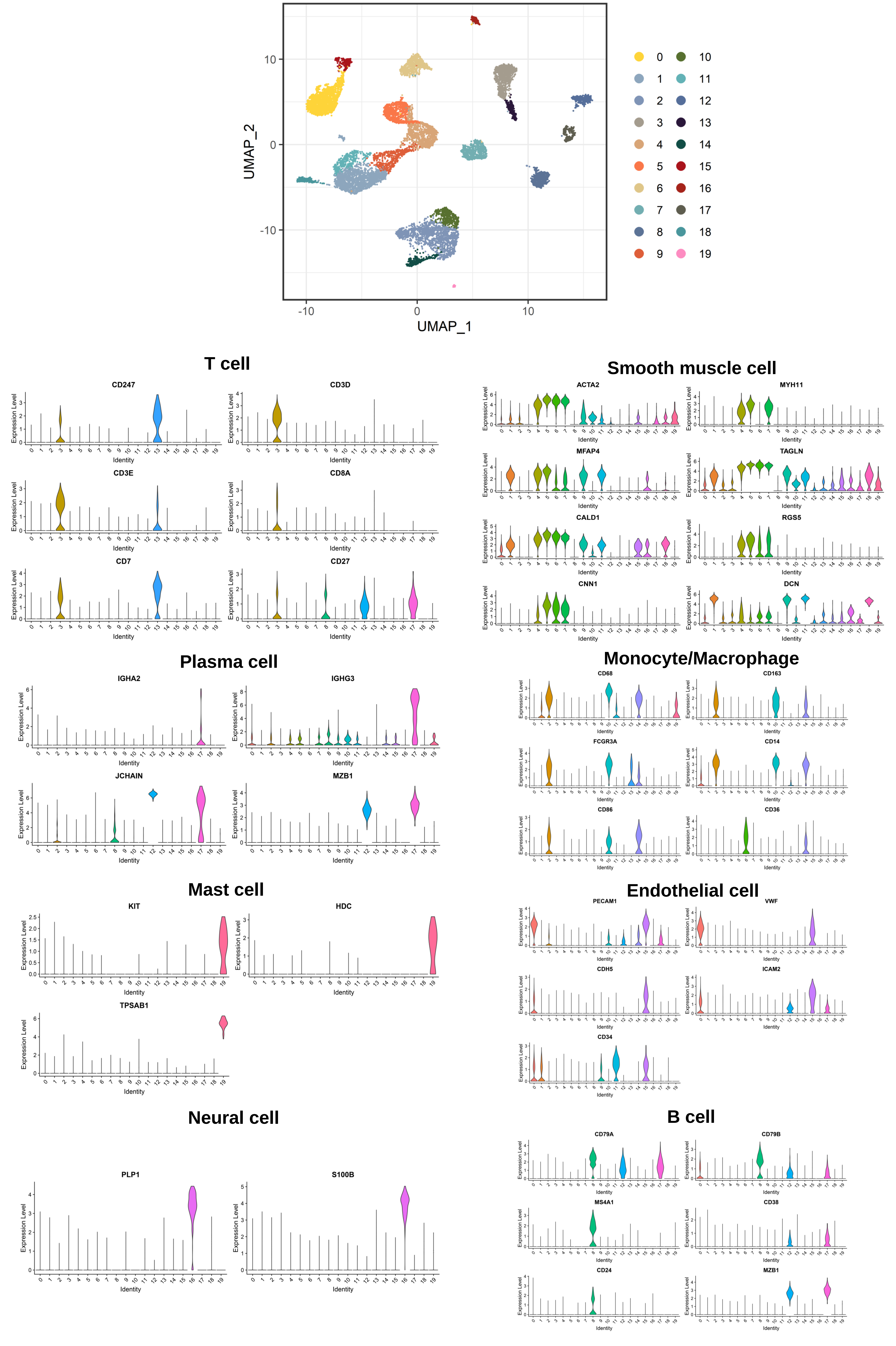
UMAP_1
UMAP_2
T cell
CD247
CD3D
CD3E
CD8A
CD7
CD27
Smooth muscle cell
ACTA2
MYH11
MFAP4
TAGLN
CALD1
RGS5
CNN1
DCN
Plasma cell
IGHA2
IGHG3
JCHAIN
MZB1
Monocyte/Macrophage
CD68
CD163
FCGR3A
CD14
CD86
CD36
Mast cell
KIT
HDC
TPSAB1
Endothelial cell
PECAM1
VWF
CDH5
ICAM2
CD34
Neural cell
PLP1
S100B
B cell
CD79A
CD79B
MS4A1
CD38
CD24
MZB1
Expression Level
Identity
